# Psychiatric manifestations in moyamoya disease: more than a puff of smoke? a systematic review and a case-reports meta-analysis

**DOI:** 10.3389/fpsyt.2024.1371763

**Published:** 2024-03-21

**Authors:** Luigi F. Saccaro, Clément Mallet, Alexandre Wullschleger, Michel Sabé

**Affiliations:** ^1^Psychiatry Department, Geneva University Hospital, Geneva, Switzerland; ^2^Psychiatry Department, Faculty of Medicine, University of Geneva, Geneva, Switzerland

**Keywords:** psychotic disorders, delusions, affective symptoms, depression, anxiety, tourette syndrome, attention deficit hyperactivity disorder, panic disorder

## Abstract

**Introduction:**

Moyamoya disease (MMD) is a life-threatening condition characterized by stenosis of intracranial arteries. Despite the frequency and the impact of psychiatric symptoms on the long-term prognosis and quality of life of MMD patients, no systematic review on this topic exists.

**Methods:**

This systematic review and meta-analysis included 41 studies (29 being case reports), from PubMed, Scopus, Embase until 27/3/2023, on MMD patients exhibiting psychiatric symptoms.

**Results:**

Despite a fair average quality of the articles, quantitative synthesis through logistic regression was possible only for case reports, due to heterogeneity between the other studies. Psychosis, the most frequent psychiatric symptom reported in case reports, was more frequent in MMD patients with left hemisphere involvement. Neurological symptoms occurrence increased the odds of MMD diagnosis preceding psychiatric symptoms. Psychiatric symptoms are highly prevalent in MMD patients and are relatively often the only presenting symptoms.

**Discussion:**

We discuss the diagnostic, therapeutic, and prognostic implications of recognizing and characterizing specific psychiatric symptoms in MMD, outlining preliminary guidelines for targeted pharmacological and psychotherapeutic interventions. Lastly, we outline future research and clinical perspectives, striving to enhance the oft-overlooked psychiatric care for MMD patients and to ameliorate their long-term outcome.

**Systematic Review Registration:**

https://www.crd.york.ac.uk/PROSPERO/, identifier CRD42023406303.

## 1 Introduction

Moyamoya disease (MMD) is a potentially life-threatening disease characterized by stenosis or occlusion of intracranial arteries. The exact cause of MMD is still unknown, but it is believed to be at least partly hereditary with mainly maternal transmission and a high female-to-male incidence ratio. The distal segments of the internal carotid arteries, along with the proximal middle and anterior cerebral arteries, are most frequently affected (1). While patients may present with unilateral stenosis, bilateral involvement can develop over time. In rarer instances, the posterior circulation may also be affected, particularly the posterior cerebral artery (2). Pathological MMD findings include gradual narrowing of the blood vessels, in the absence of major inflammation or atherosclerosis (3, 4). This progressive stenosis allows the development of compensatory networks of thin collateral vessels, after which the disease takes its name. On angiographic images, these compensatory networks resemble to “puffs of smoke”, hence the term “Moyamoya”, which in Japanese means hazy like a puff of smoke in the air. When this condition is idiopathic it is referred to as MMD. Conversely, when the vascular findings are observed alongside an associated condition, e.g. neurofibromatosis type 1, it is termed Moyamoya syndrome (MMS) (5). Both MMD and MMS share similar symptoms including transient ischemic attacks, seizures, headaches, cognitive impairments, and can result in the occurrence of ischemic or hemorrhagic stroke in both pediatric and adult populations (6). However, MMD typically presents with bilateral vascular involvement (although there may be a predominant side), whereas MMS may exhibit unilateral or bilateral vessel involvement (7). Treatment options primarily aim to restore blood flow through surgical interventions. Although MMD is less common than MMS and is considered a rare condition, its prevalence has been reported to be as high as 3.1 per 100,000 individuals in Japan, where it is most commonly observed (8, 9). MMD exhibits a bimodal age presentation, with the first peak typically occurring during the first decade of life and the second during the fourth decade (10).

Four main MMD types have been identified based on their clinical presentations: the hemorrhagic, the epileptic, the transient ischemic attack (TIA), and the ischemic infarct types (8). The hemorrhagic type is more prevalent in the fourth decade of life, and is related to intracranial hemorrhage, arising from the rupture of the delicate network of collateral vessels, primarily manifesting as intraparenchymal bleeding, although it can occasionally result in intraventricular or subarachnoid bleeding (11). The epileptic type is characterized by seizures, which have been found to be the initial manifestation in up to 34% of all cases in a prospective study of 23 MMD patients (12). The most common MMD type is the ischemic one. The ischemic infarct type accounts for approximately 95% of younger patients (first decade of life), who often suffer also from the TIA type. Especially in these two last types, it can be hypothesized that certain triggers, such as crying, coughing, blowing, hyperventilation, or stress can lead to ischemic symptoms due to reduced carbon dioxide levels and vasoconstriction (8, 13). A decrease in cerebral blood supply, and any organic cerebral insult in general, can contribute to the development of psychiatric symptoms. The localization of cerebral lesions may also be specifically linked with the type of associated psychiatric symptoms. Indeed, recent research has placed a growing emphasis on understanding and characterizing the psychiatric manifestations of MMD (14). However, there is a notable gap in evidence regarding the association between demographic characteristics, such as gender, patterns of MMD (e.g., hemispheric laterality of MMD involvement or the presence of stroke), and distinct psychiatric symptoms or diagnostic presentations. Recognizing these characteristics and associations holds the potential to improve the diagnosis of MMD in psychiatric patients, enhance the precision of psychiatric symptom screening in MMD patients, and optimize psychiatric care for individuals with MMD. Indeed, a significant proportion of patients with MMD may have psychiatric comorbidity (15), and could benefit the most from appropriate interventions that account for the peculiar etiopathogenesis of their psychiatric symptoms.

This pre-registered systematic review and meta-analysis provides the first systematic assessment and characterization of the existing literature on psychiatric manifestations in MMD patients, with the primary aim of improving clinical detection and characterization of psychiatric presentations of this rare pathology. By suggesting some potential pathophysiological hypotheses concerning the origin of psychiatric symptoms in MMD, and by discussing the diagnostic, therapeutic, and prognostic implications tied to the recognition and characterization of specific patterns of psychiatric symptoms in MMD, the review outlines a roadmap for future research and clinical directions, paving the way for the development of targeted psychiatric treatments and interventions for MMD patients.

## 2 Materials and methods

### 2.1 Search strategy and selection criteria

This Systematic Review and Meta-analysis was conducted following the Preferred Reporting Items for Systematic Reviews and Meta-analyses (PRISMA) guidelines (16), and was pre-registered in the PROSPERO database (registration number CRD42023406303).

We used a two-step approach. First, we searched PubMed (including MEDLINE), Scopus, Embase for articles published until 27/3/2023. The search strategy included intentionally broad terms: (moyamoya OR Moya moya) AND (psychiatr* OR schizo* OR psychosis OR psychotic OR delusion OR delusional OR delirium OR stress OR anxiety OR mood OR depress* OR mania OR hallucinat* OR acute confusional state OR delirium). Secondly, we manually searched the lists of the references of retrieved articles. The articles were screened by title and abstract by two independent researchers (LFS, CM) and disagreements were resolved by a third senior reviewer (MS). Duplicate references were excluded. The full texts identified were further inspected for eligibility against *a priori-*defined exclusion and inclusion criteria. The articles that remained were subjected to screening based on their titles and abstracts. Subsequently, the full texts of the identified articles were meticulously examined to determine their eligibility, in accordance with predefined exclusion and inclusion criteria.

**Figure 1:**
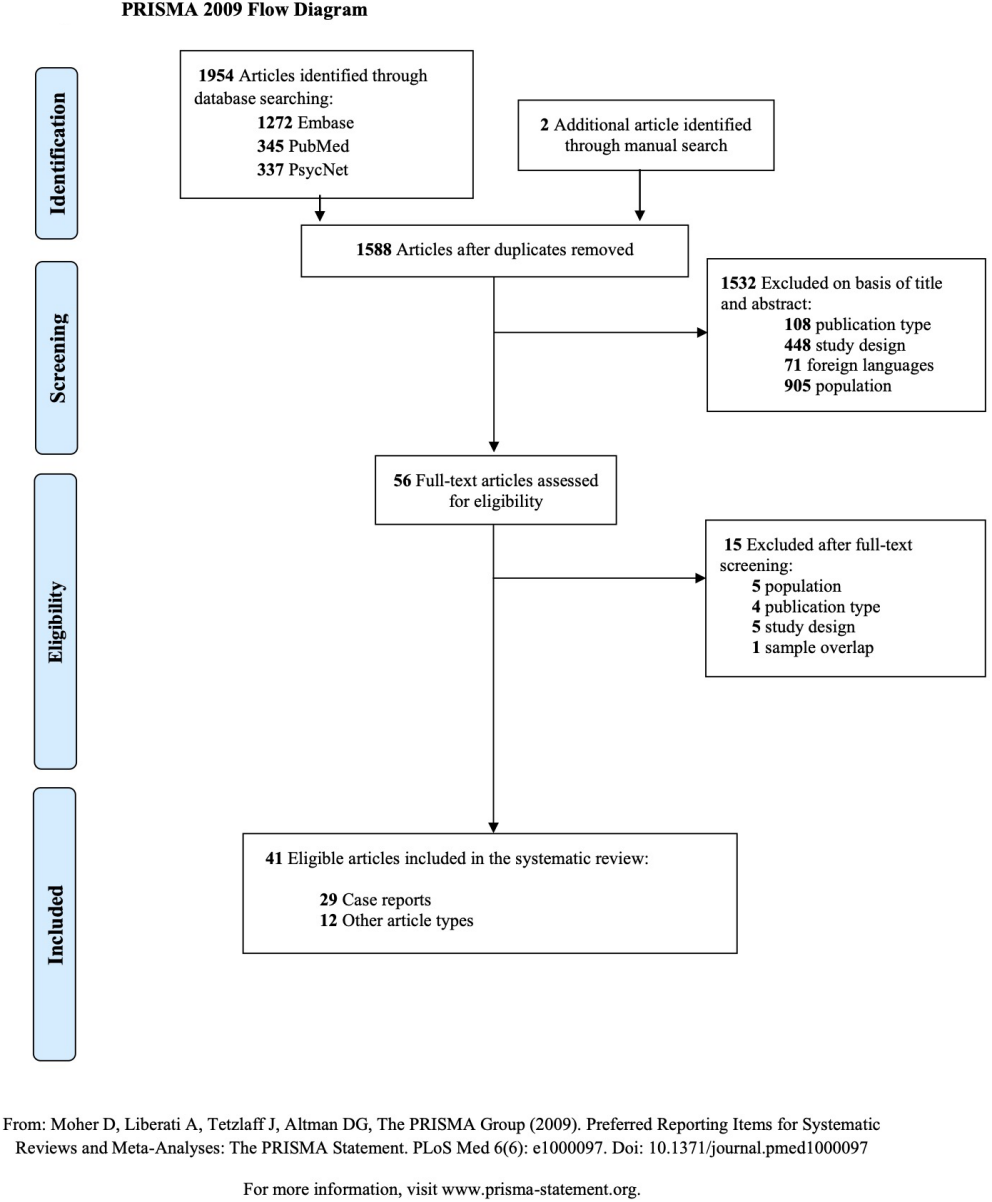
PRISMA 2009 Flow Diagram. Articles selection process.

As further detailed in **Supplementary Table 1**, we included original articles in English that met the following Participants, Interventions, Comparators, Outcomes, and Study design (PICOS) criteria. Briefly, we included papers that studied any type of MMD or MMS patients that experienced psychiatric symptoms, or psychiatric diagnoses. Any intervention, comparison, and outcome were included. We included all study designs apart from conference abstracts and presentations, reviews, meta-analyses, or systematic reviews. We excluded studies not in English, French, or Italian. The selection process was documented in the PRISMA flow diagram (**Figure 1**).

### 2.2 Data extraction

The following variables were extracted by two independent researchers (LFS, CM) from each article, when available: authors and year of publication, methodology, main statistical test used, outcome and significant findings between the variables analyzed, number of psychiatric patients and controls, number of females, mean age, patient’s laterality (ambidextrous, right- or left-handed), personal and family clinical history, lateralization of MMD involvement, localization of cerebral lesions, the diagnosis and symptoms of patients, whether psychiatric symptoms and diagnoses were adequately assessed (i.e., based on DSM or ICD criteria), age at psychiatric and neurologic symptoms onset, temporality of their appearance, Suzuki staging, information on substance abuse, author’s interpretation of the findings, follow-up information, presence of an identified stressor, effectiveness of psychotropic drugs and/or neurosurgery in improving the symptoms. Disagreements were resolved by a third senior reviewer (MS). If essential information was absent, we reached out to the corresponding author; if we did not receive a response within 3 months, we omitted the study. The authors report ethnicities as they were described in the included articles. It should be clear that this does not necessarily reflect the perspective of the authors, nor necessarily the clearest way to report ethnicities.

### 2.3 Data synthesis and analysis

We divided the relevant studies into two separate tables, one for case reports, and one for other study designs (**Supplementary Tables 2**, **3**, respectively). We used Excel and R^©^ (version 4.2.1.) to extract descriptive statistics. We calculated odds ratio (OR) through logistic regression analysis in R^©^, using the package *dplyr* (17) for the relevant variables that had less than 10% of missing values. These included the risk of psychosis depending on the laterality of the MMD involvement and the presence of stroke, the temporality of diagnosis depending on the presence of stroke and the kind of symptoms, as well as gender differences.

### 2.4 Quality assessment

The quality of the selected studies was assessed with the Newcastle-Ottawa Scale (NOS). The NOS is a widely used risk-of-bias assessment tool consisting of two sections: one for case-report studies (18) and the other for cohort studies (19). Case reports were evaluated using the NOS considering its four domains: patient selection, ascertainment of exposure, causality, and reporting. Other studies were evaluated using the NOS considering its three domains: patient selection, comparability, and exposure (**Supplementary Tables 2**, **3**). The risk of bias and concerns regarding applicability were analyzed for each domain.

## 3 Results

1588 non-duplicate studies were identified through database and manual searching and they were screened, as described in **Figure 1**. The final sample included 41 articles reporting psychiatric symptoms in MMD patients. Of these, 29 were case reports, and 12 were other kind of observational studies. No study was excluded due to missing information. All the extracted data is detailed in **Supplementary Tables 2**, **3**, and in the following paragraphs.

### 3.1 Results of case reports on psychiatric symptoms in moyamoya disease

#### 3.1.1 Descriptive results

The 29 case reports included (8, 11, 13–15, 20–43) comprised in total 29 MMD patients (15 females) (**Table 1**; **Supplementary Table 2**). Only one case of MMS was reported (24). The average age at first psychiatric symptoms was 23.27 years, the average age at first neurological symptoms was 23.56 years, and the average age at MMD diagnosis was 23.57 years. In seven cases, the MMD diagnosis preceded the psychiatric symptoms (range: 3 days to 12 years). In 19 cases, the psychiatric symptoms preceded the MMD diagnosis. The remaining 3 cases did not have information on this point.

**Table 1:** Case reports on psychiatric symptoms in moyamoya patients.

First Author Year	Psychiatric symptoms/diagnosis	Vascular Territory involved	Age at first psychiatric symptoms	Ethnicity, sex	Time between psychiatric symptoms and MMD diagnosis (positive= psychiatric symptoms first, negative= MMD diagnosis first)	Authors' interpretation	Other relevant findings
Wu et al. (43)	Delusion of persecution, AHVH	LMCA	11	NG, F	+15y	Psychosis as sole psychiatric manifestation of MMD	Left hemisphere hypoperfusion. No response to quetiapine nor aripiprazole.
Sanchez et al. (14)	Depression, fantastical delusions, AH	ICA, MCA, LACA	37	Caucasian, M	-3y	Atypical psychosis due to MMD	No relapse 2 m after stopping treatment.
Navkhare et al. (39)	Mania (psychotic)	ICA, MCA, ACA	24	NG, F	+10y	Atypical clinical presentation of mood disorder associated with MMD	Mood symptoms controlled at FU under lithium and valproate
Richards et al. (15)	Psychosis	ICA, MCA, ACA	54	Chinese, F	+3w	NG	NG
Chaughtai et al. (23)	Anxiety	LMCA, LACA	28	Caucasian, F	+21d	Anxiety as a symptoms of MMD	NG
Singh et al. (42)	Delusion of persecution	ICA, MCA, ACA	19	Asian, F	+3y	Delusions as symptoms of MMD-associated stroke	NG
Mughal et al. (37)	Depression, suicidal thoughts	NG	12	NG, F	NG	Recurrent depression as a symptom of MMD	Improvement of depression after sertraline
Gnanavel et al. (27)	Delusion of persecution , AH	LMCA	8	Asian, M	-1y	Organic psychosis retained.	No relapse at 2 w FU under treatment
Behere et al. (20)	Mania (non psychotic)	LICA, LMCA	16	NG, M	+3m	Atypical presentation due to interaction between MMD and mood disorder	No relapse at 6 m FU under treatment
Lubman et al. (35)	Psychosis	ICA, MCA, ACA	21	Vietnamese, M	+2y	Atypical presentation due to interaction between MMD and SCZ	Mild posterior parietal hypoperfusion
Lee et al. (33)	confabulation, inappropriate behavior, mood symptoms	ICA, MCA	45	NG, F	+3w	Fluctuanting frontal symptoms associated with hypoperfusion of frontal lobe during emotionally stressful situation	Partial improvement of mood with unspecified mood stabilizer
Klasen et al. (29)	Delusion of persecution, AHVH	LICA, LMCA, LACA	16	European, M	+10d	Psychotic symptoms as the sole manifestation of Moyamoya	No relapse at 1 y FU without treatment
McDade et al. (36)	Delusion of persecution , AH	NG	19	Asian, M	-12y	SCZ as symptoms of MMD-associated stroke in the dominant hemisphere	Stable at 2 y FU with treatment
D’netto and Sudarsanan (25)	Psychosis	ICA, ACA	11	Asian, M	+2m	SCZ as symptoms of MMD	Fenotiazide not effective. Stable with phenobarbitone at 1 y FU.
Nagata et al. (38)	Aggressive and labile personality change	RACA, RMCA	50	NG, M	-1w	Impulsiveness and aggressiveness resulting from right frontal lobe stroke	Stable at 1 y FU with carbamazepine.
Ghignone et al. (26)	Catatonia	ICA, MCA, RPCA	30	African American, F	+1w	Catatonia resulting from moyamoya syndrome	Stable 2 m after ECT.
McCreary et al. (8)	Autism	ICA	NG	NG, M	NG	NG	NG
Patra et al. (40)	Inattention and hyperactivity	ICA	11	NG, M	+3y	Difficulty sustaining attention due to hypoperfusion of the frontal lobe	Surgical revascularization relieved headaches, not ADHD symptoms.
Mohapatra et al. (11)	Subsyndromal inattention, emotional lability, and hyperactivity	ICA	10	NG, M	+4y	Potential association between subsyndromal ADHD symptoms and MMD	NG
Wunderle et al. (13)	Anxiety	ICA, MCA	40	NG, F	+2m	NG	Increased cerebral metabolism or oxygen consumption in response to thyroid hormones may exacerbated MMD symtpoms.
Lai (31)	Catatonic depression	ICA	33	Chinese, F	+1y	Depression as a manifestation of hemorrhagic MMD in left hemisphere	Loxapine and mirtazapine yielded no improvement, RNF213 gene mutation.
Lee et al. (32)	Psychogenic dizziness	MCA	28	Korean, M	-3y	NG	NG
Bendre (21)	Depression, anger outburst with circumstantial amnesia	NG	12	Indian, F	+2y	Depressive symptoms may be the presenting symptoms of MMD	NG
Lin et al. (34)	Panic symptoms	ICA, ACA, MCA	10	Chinese-American, M	+2y	Panic symptoms as the first MMD manifestation	NG
Schwarz et al. (41)	Depression, anxiety, self-cutting behaviors	ICA, ACA, MCA	24	Caucasian, F	-9y	NG	NG
Bose et al. (22)	Tourette's syndrome	LICA, LMCA	4.3	NG, M	+1.5y	Association of paediatric Tourette’s syndrome with MMD	NG
Gomez and Hogan (28)	History of depression	ICA	NG	NG, F	NG	NG	NG
DeDios-Stern and Ventura (24)	ADHD, separation anxiety	ACA, MCA	4	Latino, F	+1.8y	NG	NG
Koda et al. (30)	Capgras syndrome	ICA, ACA	51	NG, F	-3d	Frontal hypoperfusion due to MMD may explain the symptoms	NG

ADHD, attention deficit hyperactivity disorder; AHVH, audio/visual hallucinations; d, days; ECT, Electroconvulsive therapy; F, female; FU, Follow-up; m, months; M, male; MMD, Moya-moya disease; NA, Not applicable; NF1, Neurofibromatosis 1; NG, Not given; NOS, not otherwise specifies; SCZ, Schizophrenia; TIA, Transient Ischemic Attack; THC, cannabis; R/LICA, right/left Internal carotid artery; R/L MCA, right/left middle cerebral artery; R/LACA, right/left anterior cerebral artery; R/LPCA, right/left posterior cerebral artery; w, weeks; y, years. Ethnicity and symptoms are reported as defined in the case reports.

Psychiatric symptoms reported in the included case reports were varied (**Supplementary Table 2**). The most frequent was psychosis or delusions (41%, *n=*12, of which 5 with hallucinations, i.e. 17% of the total). In 10 of these case reports (i.e. 83% of the 12 case reports on psychotic symptoms), psychotic symptoms improved after psychotropic medications (mainly olanzapine, clozapine, and risperidone), while the remaining two articles report that olanzapine was ineffective (15, 42). Other common symptoms were anxio-depressive symptoms (*n=*11, i.e. 38%), including, in one case, panic attacks. Three case reports (i.e. 10%) reported ADHD symptoms (*n=*3). Two case reports reported catatonic symptoms.

Seven MMD patients (i.e. 24% of the total) presented isolated psychiatric symptoms, 4 (14%) presented psychiatric symptoms first, followed by neurological symptoms, 10 (34%) presented neurological signs or symptoms first, followed by psychiatric symptoms, 7 (24%) presented concomitant psychiatric and neurologic signs or symptoms (<1 month). There were no isolated neurological symptoms, in agreement with our screening criteria. 17 (58%) MMD patients presented hemorrhagic or ischemic stroke, the rest did not show evidence of neither (**Supplementary Table 2**).

#### 3.1.2 Logistic regression analysis results

Logistic regression analysis was conducted to investigate the odds ratios (OR) for various categorical variables related to psychosis and psychiatric symptoms in patients with MMD, when the number of missing values allowed it.

Firstly, we calculated the OR for experiencing psychosis in MMD patients with predominantly left hemisphere involvement (i.e., left hemisphere lesions or vascular stenosis/occlusion), compared to MMD patients with bilateral or right hemisphere involvement. The OR was 8.3 (95% CI: 1.3439 to 51.6724, p-value= 0.02). This suggests a significantly increased risk of experiencing psychosis in MMD patients with mainly left hemisphere involvement, compared to MMD patients with bilateral or right hemisphere involvement. None of the included articles reported left-handed individuals.

Secondly, we examined the OR for being diagnosed with MMD after isolated psychiatric symptoms in subjects exposed to hemorrhagic or ischemic strokes. The OR was 0.0625 (95% CI: 0.0062 to 0.633, p= 0.018). These results indicate that MMD patients exposed to hemorrhagic or ischemic strokes were significantly less likely of being diagnosed with MMD following isolated psychiatric symptoms only, compared to MMD patients without strokes.

Next, we explored the OR for receiving an MMD diagnosis before psychiatric symptoms in individuals whose neurological symptoms preceded psychiatric symptoms. The OR was found to be 8.5 (95% CI: 1.2471 to 57.9331, p-value = 0.028). This suggests that MMD patients who experienced neurological symptoms before psychiatric ones were more likely to also be diagnosed with MMD before experiencing psychiatric symptoms, compared to those who did not have neurological symptoms, had concomitant neurological and psychiatric symptoms, or had neurological symptoms after psychiatric symptoms.

As far as negative findings are concerned, MMD patients exposed to hemorrhagic or ischemic strokes did not have a different risk of experiencing psychosis than MMD patients who did not suffer strokes (p=0.46). Furthermore, gender did not play a significant role in the risk of psychosis nor of being diagnosed with MMD following isolated psychiatric symptoms (p>0.1 for both comparisons).

### 3.2 Results of other studies on psychiatric symptoms in moyamoya disease

We included 12 other studies that were not case reports nor case series (44–55). These comprised in total 1043 MMD patients (mean age: 37 years old; mean percentage of females: 58%). Two studies were retrospective (45, 46), two were prospective (50, 51), and the remaining nine were cross-sectional (**Table 2**; **Supplementary Table 3**).

**Table 2:** Other articles on psychiatric symptoms in moyamoya patients.

First Author, Year	Psychiatric symptoms/diagnosis (% prevalence)	Study type	Ethnicity (%)	Main outcome and findings	Sample size (% of females)	Mean/Median age in years
Haas et al. (45)	mood symptoms (30%) psychotic symptoms (34%) obsessive–compulsive disorder (39.3%), abnormal emotional role function (34.4%)	retrospective	Caucasian 91%, Asian 9%	Depressive symptoms significantly associated with infarctions in the right MCA territory	67 (74)	38 (median)
Wang et al. (52)	mood symptoms (NG) anxiety symptoms (NG)	cross-sectional	NG	Identification of MMD subtypes: high depression‐high anxiety‐low cognition (HE‐LC, 50%), low depression‐low anxiety‐high cognition (LE‐HC, 14%), and low depression‐low anxiety‐low cognition (LE‐LC, 36%).	39 (NG)	NG
Po et al. (50)	Sleep disturbance (3%)	prospective	Caucasian 51/65 (78%), Asian 9/65 (14%), African 5/65 (8%)	11% of MMD patients had behavioral problems at follow-up	65 (52)	7.4 (mean)
Zhao et al. (55)	mood symptoms (NG) anxiety symptoms (NG)	cross-sectional	NG	Higher depression scores increased the risk of ADL decline (p=0.001)	411 (49)	50 (mean)
Karzmark et al. (46)	mood symptoms (28%) anxiety symptoms (29%)	retrospective	white (20), Asian (5), black (3), and other (2)	High rate (37%) of depression and/or anxiety in MMD patients	30 (80)	36 (mean)
Festa et al. (44)	mood symptoms (36% minimal, 36% mild, 12% moderate, 16% severe )	cross-sectional	59% Caucasian, 10% African-American, 10% Hispanic, 21% Asian	High rate (28%) of moderate to severe depressive symptoms in MMD patients	29 (62)	40 (mean)
Li et al. (47)	mood symptoms (NG) anxiety symptoms (NG)	cross-sectional	NG	Anxiety and depression symptoms negatively correlated with the resting-state BOLD activity of the medial superior right frontal gyrus (p<0.01)	17 (82)	50 (mean)
Liu 2018	mood symptoms (46%) anxiety symptoms (50%) PTSD (47,5%)	cross-sectional	NG	High rates of clinical symptoms of depression (46.7%), anxiety (50%), PTSD (47.5%) in MMD.	120 (55)	43 (mean)
Yang et al. (53)	mood symptoms (NG) anxiety symptoms (NG)	cross-sectional	NG	In MMD patients, average HADS anxiety and HADS depression scores were 7.17 (SD 3.38) and 7.14 (SD 3.51), respectively	93 (70)	40 (mean)
Oh 2012	mood symptoms (52%) anxiety symptom (49%) personality traits (49%) Somatization (52%)	cross-sectional	NG	Higher anxiety, depression and somatization in MMD than in the HC (p = 0.014, 0.002 and 0.006, respectively).	37 (0)	19 (median & mean)
Su et al. (51)	mood symptoms (73%) anxiety symptoms (31%) Phobia, OCD (NG)	prospective	Chinese	Higher prevalence of depressive symptoms in patients with hemorrhagic MMD than in those with spontaneous IVH (p< 0.005).	26 (46)	44 (mean)
Yang et al. (54)	mood symptoms (NG) anxiety symptoms (NG)	cross-sectional	NG	MMD patients who had been diagnosed with the disease for less than a year had significantly greater depression than those diagnosed for a year or more (P = 0.039).	109 (71)	42 (mean)

ADL, Activities of Daily Living; BOLD, Blood Oxygenation Level Dependent MMD, Moyamoya Disease; MCA, Middle Cerebral Artery; NG, not given; HADS, Hospital Anxiety and Depression Scale ; HC, Healthy controls ; IVH, intraventricular hemorrhage; PTSD, Post-Traumatic Stress Disorder.

The most commonly reported psychiatric symptoms in MMD patients were anxio-depressive symptoms. Mood symptoms (exclusively depressive symptoms) were reported by all (*n=*11) but one study (50), which was on pediatric patients. The average percentage of MMD patients experiencing depressive symptoms, among the five studies that reported it, was 46%, (range 28% to 73%). The average percentage of MMD patients experiencing anxiety symptoms, among the four studies that reported it, was 40%, ranging from 29% to 50%. Only one study reported that 34% of 67 MMD patients presented psychoticism traits (45). Two studies (45, 51) reported OCD symptoms, while one study reported a high prevalence (47.5%) of PTSD symptoms in 120 MMD patients (48).

Only one study gave detailed information on the brain regions involved (44). Not enough studies included detailed information on the laterality and ethnicity of MMD patients, or on the percentage of MMD patients that had a clinical history of stroke, transient ischemic attack, or of psychiatric disease. No study provided information on substance use or abuse, nor on the effectiveness of neurosurgery or psychotropic medication on psychiatric symptoms in MMD patients (**Supplementary Table 3**).

The lack of data and heterogeneity of methods and populations hampered the realization of a quantitative synthesis of these studies.

### 3.3 Quality assessment

The results of the methodological quality assessment through the NOS are reported in **Supplementary Tables 2**, **3**.

#### 3.3.1 Quality assessment of other studies on psychiatric symptoms in moyamoya disease

Overall, the average quality of the case reports included was fair (5.5/10, a case report is considered valid if above 5/10). However, it should be noted that since a dose-response assessment and challenge-dechallenge-rechallenge protocols are not applicable to life-threatening, structural, disorders such as MMD, we automatically assigned one point for each of those criteria (**Supplementary Table 2**).

#### 3.3.2 Quality assessment of case reports on psychiatric symptoms in moyamoya disease

The average quality of the other studies was also fair (5/9), all studies assessed the exposure (MMD) and the outcome (psychiatric symptoms) in mostly representative cohorts. The majority of the studies (65%) reported that psychiatric symptoms were assessed according to DSM or ICD criteria. However, no study demonstrated that the outcome of interest was not present at the start of the study, and only a fourth of the studies actually included an adequate follow-up to monitor psychiatric symptoms over time. This is due to the fact that very few studies focused on psychiatric symptoms, and psychiatric scores were mostly collected to exclude confounding while evaluating other variables, such as neurocognitive impairment or quality of life (**Supplementary Table 3**).

## 4 Discussion

This pre-registered systematic review and meta-analysis investigating, for the first-time, psychiatric comorbidity of MMD yielded 41 relevant studies, of which 29 were case reports. The quality of the included studies was generally fair with a low risk of bias.

Notwithstanding some limitations detailed in Section 4.1, both quantitative and qualitative syntheses of our systematic review and meta-analysis highlight that psychiatric symptoms are highly prevalent in MMD patients, and that relatively often these symptoms are the only presenting symptoms leading to an MMD diagnosis. In such cases, none of the patients described in the included case reports had a prior psychiatric history. These findings are in line with two previous narrative reviews (14, 15) and emphasize the vital significance of improving recognition and care of psychiatric symptoms in individuals with MMD, as their diagnosis and management can be particularly challenging. To strive towards this goal, we cautiously propose some preliminary hypotheses on the characteristics of psychiatric symptoms in MMD, clinical implications, and potential psychotropic treatments that have shown promise in managing psychiatric symptoms in MMD patients, based on the findings of our review.

The review did not highlight gender differences in psychiatric symptoms in MMD patients. However, our included sample demonstrated a marginal preponderance of females, in agreement with the well-documented higher prevalence of MMD in women (56). Concerning age differences, qualitative synthesis suggests that anxio-depressive symptoms are more common in adults, while, in children and adolescents, psychosis, ADHD symptoms, emotional lability, and impaired concentration with poor academic performance have been described.

The psychiatric symptom most frequently described in the case reports was psychosis, while in other types of studies anxio-depressive symptoms were the most common ones. This may reflect a reporting bias among case reports, as well as the fact that psychotic manifestations are usually more striking and temporally better defined than anxio-depressive ones and may thus be linked more easily with MMD. Notwithstanding these considerations, intriguing patterns emerge from the review. We will discuss them in the following section, although further, mechanistic, research is needed on these topics, as mentioned in the Limitations (Section 4.2.).

First of all, the fact that psychotic symptoms were more common in MMD patients with predominant involvement of the left hemisphere (i.e. the dominant one, considering that none of the included articles reported left-handed individuals), suggests a potential association between the location of MMD pathology and specific psychiatric manifestations. The left hemisphere is prominently involved in language processing, logical reasoning, and semantic memory. Thus, damage to this region could contribute to cognitive distortions and impaired reality testing, which are often observed in individuals with psychosis, delusions, or schizophrenia (57). For instance, we may speculate that lesions affecting Broca’s area or the left temporoparietal junction may disrupt the seamless integration of language and social cues, leading to misinterpretations and distorted beliefs characteristic of psychotic thinking (58). Furthermore, the left hemisphere plays a pivotal role in top-down cognitive control and attentional processes. Lesions or reduced blood flow in this region may compromise an individual’s ability to filter out irrelevant stimuli and maintain focused attention. As an hypothesis, this may potentially lead to aberrant saliency assignment, which is one of the proposed physiopathological mechanisms for psychosis since increased susceptibility to external stimuli being misinterpreted or distorted fuels the development of delusions or other psychotic phenomena (59, 60). In fact, a recent fMRI study highlighted aberrant functional connectivity between the saliency network and the left, but not the right, hippocampus in bipolar disorder, which may represent a marker of vulnerability to psychosis (61). Similarly, Liu and colleagues (62) highlighted structural and functional alterations of the left thalamus in schizophrenic patients, and these impairments correlated with symptoms. Thalamus involvement in psychosis is, indeed, well-established and supported by fMRI studies (63). Finally, a systematic review on neural underpinnings of delusional symptoms in schizophrenia and Alzheimer’s disease patients reported lateralized findings of gray matter reductions in the left claustrum (64), reinforcing the notion of left hemisphere involvement in the genesis of psychotic symptoms.

While these observations align with the hypothesis of left hemisphere involvement in psychotic symptoms in some populations, a systematic review in patients with post-stroke psychosis found that right hemisphere lesions were more common (in around 40% of the cases, against 11% with a different pattern of lesions). However, no quantitative synthesis was performed in this systematic review, and about half of the included case reports did not give information on lesions localization, limiting the reliability of these results (65). Considering our finding of an increased association of psychosis with left hemisphere lesions in MMD patients, it may be hypothesized that psychosis associated with MMD has different characteristics and mechanisms compared to poststroke psychosis, even in the presence of brain lesions.

This hypothesis is rooted in the proposed physiopathology of the generation of psychiatric symptoms, and specifically of psychotic outbursts in MMD. Indeed, as mentioned in the Introduction, it has been proposed that these are most often acute or acutely exacerbated, commonly in response to certain triggers, such as crying, coughing, blowing, hyperventilation, or stress. These can lead to reduced carbon dioxide levels and vasoconstriction, which is more deleterious in the brain regions already significantly affected by MMD (8, 13). A consequent transitory decrease in cerebral blood supply, especially in such vulnerable regions, may contribute to the development of panic (34, 66) or even psychotic symptoms, which have been consistently associated with cerebral hypoperfusion (67). On the other hand, in post-stroke psychosis, the affected brain regions are permanently impaired, and the symptoms may result from more stable reorganization of brain networks or neurotransmitters imbalance. This hypothetical mechanism gains further credence from the observation that electroencephalograms in MMD patients, particularly among pediatric patients, frequently reveal persistent build-up and rebuilding phenomena. This phenomenon entails the continued presence of high-amplitude slow waves beyond 30 seconds following hyperventilation. Notably, these EEG changes often normalize post-surgery and show a strong correlation with cerebral ischemic events and vascular reserve, a link substantiated by various imaging techniques such as cerebral angiography or single-photon emission-computed tomography (10, 34, 68). Indeed, the context of the occurrence of psychotic symptoms in some of the included case reports support the existence of a link between certain triggers that may lead to cerebral vasoconstrictions and psychotic symptoms. For instance, Klasen et al. (29) suggest a causal link between MMD and psychotic symptoms in a 12-year-old boy, given the improbability of these two events co-occurring independently at his age, without any previous family or personal psychiatric history, the specific involvement of the left hemisphere and left basal ganglia, and the physical exertion accompanied by hyperventilation preceding the psychosis outburst. This is in agreement with another case report describing a woman who presented recurrent exacerbation of psychotic symptoms and hallucinations after physical exercise for a decade before being diagnosed with MMD, and before finally responding to clozapine (43). Of note, her pattern of cerebral hypoperfusion was clearly predominant in the left hemisphere (43). Concerning hallucinations in particular, another case report (14) argues that they were linked to hypoperfusion in the territory of the posterior cerebral arteries. This hypothesis aligns with the observation that psychosis appears to be a more frequent manifestation in children and young adults with MMD (15), who more often present with the ischemic/TIA type of MMD, potentially exposing them to a higher likelihood of psychotic outbreaks in association to transitory exacerbations of cerebral hypoperfusion. However, MMD patients may develop psychiatric symptoms as a result of MMD at any point of their illness. In fact, insight from longitudinal studies underscores the evolving susceptibility of brain regions to hemodynamic insufficiency as MMD progresses, with a shift observed from inner to outer cortical structures and from anterior to posterior territories. This dynamic evolution potentially contributes to the development of new psychiatric manifestations over time, even in otherwise stable MMD (69).

Undoubtedly, MMD exposes patients to a myriad of other factors that may increase their risk of developing psychiatric symptoms. While our focus revolves around symptom characterization and mechanistic details are beyond the scope of this review, it is noteworthy to mention, that higher levels of stress and traumatic experiences may significantly elevate the risk of anxio-depressive disorders. The independent effect of stroke should also be considered. Indeed, stroke has been associated with different psychiatric disorders, such as depression, potentially due to serotoninergic disturbances (70), and, rarely, psychosis (65).

Frequent cerebral ischemic and hemorrhagic lesions in MMD may partially contribute to the augmented vulnerability of these patients to psychiatric symptoms. However, almost half of MMD patients with psychiatric symptoms described in the included case reports did not exhibit stroke nor TIA, aligning with previous literature (15). Additionally, our analysis demonstrates that MMD patients who experienced strokes did not exhibit a different propensity for psychosis compared to those without strokes.

Finally, we showed that MMD patients exposed to stroke were less likely of being diagnosed with MMD following isolated psychiatric symptoms only, and that the presence of neurological symptoms made it more likely for the MMD diagnosis to precede psychiatric symptoms. This pattern is logical, as MMD is often more easily diagnosed following strokes or neurological symptoms, which is not the case for instances where MMD presents solely with isolated psychiatric symptoms (15).

### 4.1 Perspectives: clinical implications of a fifth moyamoya disorder type

Considering the high prevalence of psychiatric symptoms in MMD, the not-so-uncommon occurrence of psychiatric symptoms as the presenting symptom of MMD, and the vital importance of recognizing early the underlying MMD in these cases, we argue for the introduction of a fifth MMD type, i.e., MMD with isolated psychiatric presentation, besides the classical four types described in the Introduction.

Establishing such a nomenclature is not merely symbolic, but has vital clinical implications, first of all diagnostic, therapeutic, and prognostic.

Concerning diagnosis, better characterization of patterns of presentation of psychiatric symptoms in MMD patients holds dual import: augmenting the diagnostic acumen for MMD *per se*, while concomitantly ameliorating the recognition of often-overlooked psychiatric symptoms. For instance, evidence from this review supports the importance for all clinicians of investigating anxio-depressive symptoms in MMD patients, independently of the presence of stroke. Additionally, as discussed, left hemisphere hypoperfusion may represent a marker of psychosis risk in MMD. Finally, early engagement in psychotherapy is strongly advised, taking into account the psychological precipitants identified in our review of epidemiological studies, related to decrements in quality of life, awareness of MMD prognosis, and frustration from potential cognitive impairment. While the causal relationship between MMD and psychiatric symptoms cannot be established, such a proactive approach is crucial due to its potential impact on prognosis.

Vice versa, psychiatrists should be wary of patients that, for example, present transient exacerbation of their symptoms after physical activity, in combination with atypical features, such as age of onset, no psychiatric family history, or the presence of visual hallucinations, depending on the disorder. While cerebral CT is already recommended for atypical psychiatric presentation, the aforementioned elements may prompt the evaluation of MRI angiography instead, no matter the patients’ ethnicity (15).

Evidence on therapies and optimal psychiatric care in MMD patients is vastly insufficient. This systematic review suggests that classical psychotropic medications is effective, but likely underutilized. The fact that in the vast majority of retained case reports psychotic symptoms improved after antipsychotic treatment (in particular risperidone and clozapine), suggest that these may represent an effective symptomatic treatment. Clozapine should not be considered as a first-line treatment due to its well-known side effects, and paliperidone would be expected to be effective as well, being risperidone active metabolite. However, serious concerns about the under-representation of poor outcomes remain, as discussed in the limitations below. Because untreated or poorly treated psychiatric symptoms may have a negative impact on quality of life, disability, and prognosis, a team-based multidisciplinary approach is highly recommended, with collaboration between psychiatry and neurology or neurosurgery early in diagnosis.

Two case reports described post-surgical remission (22) or improvement (24) of psychiatric symptoms of Tourette’s disorder in MMD and ADHD in MMS, respectively. While evidence concerning the effectiveness of neurosurgical treatment on psychiatric symptoms in MMD is lacking, it is very interesting to speculate that surgery may represent a promising option to treat resistant psychiatric symptoms that may be more clearly dependent on regional hypoperfusion due to MMD, such as in the case of Tourette’s disorder symptoms, which were most likely associated with hypoperfusion in the basal ganglia. As a perspective, future studies should investigate whether surgical restoration of normal perfusion may be a treatment option in cases of recurrent and antipsychotic-resistant psychotic breaks associated with predominant left-hemisphere hypoperfusion.

Lastly, in terms of prognostic implications, it is imperative for forthcoming research to concentrate on a rigorous quantification of the impact of psychiatric comorbidity and symptoms in MMD. These factors undoubtedly wield significant potential to detrimentally affect the prognosis of MMD patients. Furthermore, the effectiveness of clinical care is likely to be time-dependent, meaning that earlier or preventive interventions, along with comprehensive psychoeducation, may hold better promise for yielding more favorable and enduring outcomes for long-term mental well-being. For instance, patients and families should be instructed on the recognition of prodromal or early psychiatric symptoms, and early defusing and debriefing may be considered in vulnerable patients, who experienced particularly traumatic manifestations of MMD. Indeed, one of the included studies highlighted that MMD patients may be at risk for more severe depression in the first year after their diagnosis (54). Interestingly, another of the included study proved the feasibility of Ecological Momentary Assessment of mood in patients with MMD (53), in line with existing research on the potential of portable monitoring of mood symptoms in psychiatry patients (71).

### 4.2 Strengths, limitations, and conclusions

The primary strengths of this systematic review and meta-analysis include the fact that it fills an important gap in literature, being the first on the topic, the broad search criteria ensuring high sensitivity, the precise PICOS criteria ensuring specificity, overall adherence to PRISMA guidelines (requiring, for instance, a priori, pre-registered methods, a systematic search for eligible studies across multiple databases, blinded duplicate screening and data extraction, quality assessment), the clinical focus, and the extraction of more than 30 variables for each included study with careful reporting of not-given values so as to further highlight the gaps in the existing literature.

One main limitation was the reliance on a majority of case reports, which, while informative, inherently comes with certain limitations, such as publication bias, variable reporting quality, and a potential lack of generalizability. The review of non-case report studies was hindered by significant heterogeneity and missing data, making it challenging to draw definitive conclusions from those sources. Additionally, these studies were mainly focused on neurocognitive assessments in MMD patients, and psychiatric symptoms were mostly assessed superficially, as confounding variables. Considering the limited available data and the nature of the studies, caution is therefore necessary when interpreting these findings. While the exclusion of articles in Japanese may constitute a limitation due to the high prevalence of MMD in Japan, a targeted screening of the titles and abstracts of all the articles excluded due to foreign language (recapitulated in **Supplementary Table 4**) confirmed that these articles are not relevant to the topic of the present systematic review and meta-analysis (i.e. none of them discusses psychiatric manifestations of MMD). Finally, among the reviewed articles, only one study (51) utilized the Suzuki staging system to systematically report the pattern of MMD vascular involvement. This observation highlights the need for more consistent and systematic assessments of MMD progression and vascular involvement. A standardized approach would enable confirmation of potential patterns that might be associated with psychiatric manifestations in MMD patients.

Overall, further research is indispensable to unravel the precise mechanisms and establish a more comprehensive understanding of the pathophysiological underpinnings of psychiatric manifestations in MMD patients. Future studies should aim to employ rigorous methodologies, larger sample sizes, and standardized assessment tools to enhance the reliability and generalizability of the results. Besides, further studies specifically targeted at cross-sectional and longitudinal assessment of psychiatric symptoms and psychiatric treatment in MMD are warranted.

Despite these limitations, this systematic review and meta-analysis provides insights into the characteristics of psychiatric manifestations in MMD, and it further contributes to the existing literature by highlighting the high prevalence of psychiatric symptoms in MMD patients and the importance of recognizing them and their specific patterns or risk factors. Early detection, comprehensive evaluation of psychiatric symptoms, and adequate interventions may lead to improved diagnosis, better outcomes, and enhanced quality of life for patients affected by this life-threatening disease.

## Data availability statement

The original contributions presented in the study are included in the article/**Supplementary Material**. Further inquiries can be directed to the corresponding author.

## Author contributions

LS: Conceptualization, Data curation, Formal analysis, Investigation, Methodology, Software, Visualization, Writing – original draft, Writing – review & editing. CM: Writing – review & editing, Data curation. AW: Conceptualization, Supervision, Writing – review & editing. MS: Conceptualization, Data curation, Supervision, Writing – review & editing.

## Supplementary material

Supplementary Table 1 PICOS criteria.

Supplementary Table 2 Data Extraction for Case Reports and Case Series.

Supplementary Table 3 Data Extraction for other studies.
