# Supplementary material for: Psychiatric manifestations in moyamoya disease: more than a puff of smoke? a systematic review and a case-reports meta-analysis
Source: Front Psychiatry. 2024 Mar 21;15:1371763. doi: 10.3389/fpsyt.2024.1371763 (PMC10995700; doi:10.3389/fpsyt.2024.1371763)
Supplement: Supplementary Table 1 — PICOS criteria. [file DataSheet_1.zip › Supplementary Table 1.DOCX]

**Supplementary Table 1**. Search strategy according to the Population, Intervention, Comparison, Outcomes and Study Design (PICOS) model

| Parameter | Inclusion criteria | Exclusion criteria |
| --- | --- | --- |
| Population | At least some subjects included are persons with MoyaMoya disease (MMD) or syndrome that have experienced or present a psychiatric diagnosis or psychiatric symptoms. | - Absence of MMD.  - Absence of psychiatric diagnosis, signs, or symptoms. |
| Interventions | NA | NA |
| Comparison | Any comparison (e.g. MMD patients presenting psychiatric symptoms vs those not presenting psychiatric symptoms; MMD patients presenting psychiatric symptoms vs psychiatric patients without MMD; before/after interventions…) | NA |
| Outcomes | Prevalence and characteristics of psychiatric symptoms in MMD patients | NA |
| Study design model | All types of studies will be considered for inclusion (e.g. case reports, case series, retrospective, prospective, case- control studies, cross-sectional, and clinical trials). | Reviews, systematic reviews, metanalyses, poster and conference presentations will be excluded.  We will exclude studies not in English, French, or Italian. |
