# Supplementary material for: Psychiatric manifestations in moyamoya disease: more than a puff of smoke? a systematic review and a case-reports meta-analysis
Source: Front Psychiatry. 2024 Mar 21;15:1371763. doi: 10.3389/fpsyt.2024.1371763 (PMC10995700; doi:10.3389/fpsyt.2024.1371763)
Supplement: Supplementary Table 1 — PICOS criteria. [file DataSheet_1.zip › Supplementary Table 4.DOCX]

**Supplementary Table 4**. Articles excluded due to foreign languages.

| **TITLE** | **YEAR** | **LANGUAGE** |
| --- | --- | --- |
| Quality of life of older people with depression and dependence: validity of the SF-12 (short form health survey) questionnaire | 2021 | Spanish |
| Effects of the COVID-19 confinement on mental health among higher education students in Chile | 2021 | Spanish |
| Level of radicular stress in upper premolars and pressure exerted in adjacent tissues: a 3D mechanical model | 2021 | Spanish |
| History of child abuse among patients with bipolar disorders | 2020 | Spanish |
| Prevalence, risk factors and clinical characteristics of infantile cerebral palsy | 2019 | Spanish |
| Serum biochemical profile of Nile tilapias (Oreochromis niloticus) bred in net cages during summer and winter | 2018 | Portuguese |
| Thyroid storm precipitated by intracranial surgery in a woman with no history of thyroid disease | 2016 | Japanese |
| A Case of Ruptured Peripheral Cerebral Aneurysm at Abnormal Vessels Associated with Middle Cerebral Artery Stenosis:Similarity to Moyamoya Disease | 2016 | Japanese |
| [RN4CAST Study in Murcia: Hospital organizational characteristics and nursing staff profiles] | 2013 | Spanish |
| Syncope | 2012 | Spanish |
| Moyamoya disease | 2012 | German |
| The role of bypass surgery in the endovascular era of brain aneurysm treatment | 2011 | German |
| Internal attribution of outcome moderates the cortisol response to a cooperative task in women | 2011 | Spanish |
| Use of pacifiers and breastfeeding | 2011 | Spanish |
| Neurobiology of child abuse: The 'cycle of violence' | 2011 | Spanish |
| Antioxidant role of vitamin E in atherogenesis induced by hyperfibrinogenemia | 2010 | Spanish |
| Moyamoya disease - A rare vasculopathy in Europeans | 2010 | German |
| The genetics of human violence | 2010 | Spanish |
| Comorbidity in pathological gambling: Clinical variables, personality and treatment response | 2009 | Spanish |
| A 12-week, placebo-controlled study (6002-US-006) of istradefylline in Parkinson disease | 2009 | Spanish |
| Oxidative stress markers in atherogenesis induced by hyperfibrinogenemia | 2009 | Spanish |
| Current knowledge on the genetic factors involved in moyamoya disease | 2008 | Japanese |
| Moyamoya disease | 2008 | Japanese |
| Adult case of moyamoya disease with intractable epileptic seizures | 2007 | Japanese |
| Reevalation and appliment of cerebral revascularization | 2007 | Chinese |
| Regarding the ABP Technical Commission's document entitled "Fashion Industry Guidelines'" | 2007 | Portuguese |
| Emergent biological markers of cardiovascular risk in occupational poblation | 2006 | Spanish |
| Update in cardiac imaging techniques. Echocardiography and magnetic resonance imaging | 2006 | Spanish |
| Utility of the Multidimensional Alcohol Craving Scale (MACS) in the clinical practice | 2006 | Spanish |
| Prognostic value of valsalva maneuver-induced change in Doppler-detected ventricular filling in patients with systolic dysfunction | 2005 | Spanish |
| Echocardiographic assessment of asynchrony | 2005 | Spanish |
| Aplasia of the right internal carotid artery presenting with callosal hemorrhage: Case report and review of the literature | 2005 | Japanese |
| Primary hyperhidrosis and anxiety: A prospective preoperative survey of 158 patients | 2005 | Spanish |
| Current situation in the treatment of vasomotor syndrome | 2005 | Spanish |
| Reliability and validity of Bulimic Investigatory Test, Edinburgh (BITE) in a simple of Spanish adolescents | 2004 | Spanish |
| Specific features of pathogenesis and diagnosis of hemorrhagic stroke in young patients | 2004 | Russian |
| Temperature and death in old people | 2004 | Spanish |
| Non-ST elevation acute coronary syndrome due to an acute lesion in the left main coronary artery. Report of 6 cases | 2004 | Spanish |
| Obesity in women | 2003 | Spanish |
| Analysis of clinical differences between bulimia nervosa and sub-threshold bulimia | 2003 | Spanish |
| Primary hyperhidrosis: Prospective study in 338 patients | 2003 | Spanish |
| Personality disorder and comorbidities in a case of eating disorder of long duration | 2001 | Portuguese |
| Child abuse: Population characteristics and risk factors at the Colsubsidio welfare clinic in Bogota. ("Clinica del Buen Trato") | 2001 | Spanish |
| Chronic low back pain: multispecialty assessment of 100 patients | 2000 | Spanish |
| Study of developmental consequences of physical negligence in Spanish preschool children | 2000 | Spanish |
| An autopsy case of bilateral carotid artery occlusion with repetitive epilepsy and brain atrophy in a senile patient | 2000 | Japanese |
| Usefulness of an anesthesia information sheet before preoperative visits | 2000 | Spanish |
| Psychosomatic alterations in the international glossaries for illnesses | 1999 | Spanish |
| Puerperal depression. Related factors | 1997 | Spanish |
| Organic mental disorder in psychiatric consultation-liaison in practice | 1996 | Spanish |
| A case of cerebral aneurysm located at the leptomeningeal artery associated with occlusion of the middle cerebral artery | 1995 | Japanese |
| Propofol in continuous perfusion for anesthesia during experimental surgery in the rabbit | 1995 | Spanish |
| Middle cerebral artery occlusion with unilateral moyamoya like vessels and with ruptured anterior cerebral artery aneurysm. Its relation to be antiphospholipid antibody syndrome | 1994 | Japanese |
| Analysis of the psychiatric interconsultation demand in a General Hospital | 1994 | Spanish |
| Preoperative sedation for childhood moyamoya disease - Clinical evaluation of rectally administered midazolam | 1994 | Japanese |
| Psychiatrics emergencies, clinical and demographic characteristics in a Provincial Psychiatric Center | 1994 | Spanish |
| Neurological manifestation of Takayasu's arteritis | 1992 | Chinese |
| Pathological and clinical features of primary ventricular hemorrhage due to moyamoya disease | 1991 | Chinese |
| Influence of patient's personal characteristics on anxiety level, oral hygiene, pain intensity at infiltrative anesthesia puncture and time of professional care | 1991 | Spanish |
| Prognostic significance of silent ischemia in the exercise test in patients with coronary disease | 1990 | Spanish |
| The oncological pediatric patient: Psychology and childhood cancer | 1989 | Spanish |
| General and psychopathologic aspects of sexual abuse in children | 1989 | Spanish |
| Moyamoya syndrome in neuroanesthesia | 1987 | German |
| Immunological aspects of cerebrovascular disease | 1986 | Chinese |
| A case of moyamoya disease complicated by a depressive syndrome | 1981 | Polish |
| The pathology and clinical features of the abnormal vascular network at the base of the brain- report of 3 cases with post-mortem examination (author's transl) | 1980 | Chinese |
| Clinical and radiological analysis of the abnormal vascular network of the base of the brain | 1980 | Chinese |
| Occlusion of circulus arteriosus of Willis- report of 40 cases (author's transl) | 1980 | Chinese |
| Moyamoya disease caused by leptospiral arteritis (author's transl) | 1980 | Chinese |
| Cranio-encephalic closed traumatisms and seric levels of dopamine-beta-hydroxylase (DBH). (Possibilities of ulcers by stress) | 1979 | Spanish |
| Acute hemisyndrome in childhood | 1977 | German |
